# Review: Mixed-Matrix Membranes with CNT for CO_2_ Separation Processes

**DOI:** 10.3390/membranes11060457

**Published:** 2021-06-21

**Authors:** Marquidia J. Pacheco, Luis J. Vences, Hilda Moreno, Joel O. Pacheco, Ricardo Valdivia, Celso Hernández

**Affiliations:** 1 National Institute for Nuclear Research (ININ), Carr Toluca-Mexico s/n, Ocoyoacac 52750, Mexico; marquidia.pacheco@inin.gob.mx (M.J.P.); lvencesr@toluca.tecnm.mx (L.J.V.); ricardo.valdivia@inin.gob.mx (R.V.); 2 Technological National Institute of Mexico/Technological Institute of Toluca, Av. Tecnológico s/n, Metepec 52149, Mexico; chernandezt@toluca.tecnm.mx

**Keywords:** MMM, CNT, permeability, CO_2_ separation

## Abstract

The membranes’ role is of supreme importance in the separation of compounds under different phases of matter. The topic addressed here is based on the use of membranes on the gases separation, specifically the advantages of mixed-matrix membranes (MMMs) when using carbon nanotubes as fillers to separate carbon dioxide (CO_2_) from other carrier gas. MMMs consist of a polymer support with additive fillers to improve their efficiency by increasing both selectivity and permeability. The most promising fillers in the MMM development are nanostructured molecules. Due to the good prospects of carbon nanotubes (CNTs) as MMM fillers, this article aims to concentrate the advances and developments of CNT–MMM to separate gases, such as CO_2_. The influence of functionalized CNT or mixtures of CNT with additional materials such as zeolites, hydrogel and, graphene sheets on membranes performance is highlighted in the present work.

## 1. Introduction

The massive emission of greenhouse gases, especially carbon dioxide (CO_2_), has a serious impact on the natural environment; consequently, the capture and storage and or treatment of CO_2_ become crucial to minimize its damages on nature [1,2,3,4]. In current separation methods, such as cryogenic distillation, absorption and adsorption, the gas-separation membrane technology has gained significant importance due to its lower energy consumption, a reduced footprint, a reduced amount of capital investment, greater energy efficient and environmental viability [5,6,7,8,9].

The membranes are categorized into metallic, inorganic and polymeric [10]. Metallic membranes have excellent performance, but the cost of precious metals significantly influences the membrane selection. Inorganic membranes are good alternatives, as they have better chemical stability with lower fabrication cost. Nevertheless, a high temperature, from 200 to 900 °C, is needed to operate inorganic membranes [11]. Nowadays, polymeric membranes dominate the industry because of the outstanding economy and competitive performance. These polymeric membranes can be operated at ambient temperature and they have good mechanical and chemical properties; however, they have limited efficiency [12,13]. To overcome those disadvantages, mixed-matrix membranes (MMMs) have been technologically advanced, and they are heterogeneous membranes with additive fillers dispersed in a polymer base. In particular, MMM combines the high thermal and chemical stability of inorganic membranes and the mechanical strength of polymeric membranes at moderate operating costs.

MMMs, have a unique structure, surface chemistry and mechanical strength. When fillers (dispersed phase) are added to the polymer matrix (continuous phase), the properties of these membranes become better than polymeric membranes [14,15,16,17]. Generally, MMMs are easier to prepare and process, they are commonly synthesized by dispersing a polymer solution into the inorganic phase, and, by evaporating the solvent, a dense membrane is obtained. The MMMs have higher gas permeability and selectivity, presenting good mechanical and chemical properties [18], allowing them to be easily scaled up to an industrial level and to be straightforwardly commercialized [19,20].

A separation membrane acts as a thin interface typically in the range of 1 nm to a few microns that functions as a selective separation barrier in two phases. Generally, the intrinsic gas permeation and separation property of the selective-layer material are the main elements of the gas-separation membrane performance [21,22,23,24]. A gas-separation event involves the partial separation of a mixture of two or more components with a membrane acting as a semi-permeable barrier that allows one component to freely permeate the membrane, while delaying the permeation of other components [25,26].

Membrane separation has been recognized as a viable and effective technology at both, laboratory and industrial scale; therefore, processes involving them are rapidly growing, and they have attracted remarkable attention as the major paradigm for separation processes in key areas. A higher selectivity has a positive impact on the separation efficiency and operating costs, whereas higher fluxes lead to lower membrane area requirements and, therefore, lower general costs of the membrane system [27].

Concerning the separation performance of polymeric membranes, it is generally limited by the speed at which any compound penetrates through a membrane, this depends on a thermodynamic factor and a kinetic factor [28,29]. Typically, the properties of a gas-separation membrane are characterized in terms of gas permeability and selectivity, respectively, represented by Equations (1) and (2) [30].

The permeability (*P*) is the rate of passive diffusion of molecules through the membrane and is defined as follows:

(1) $$P=Q\frac{l}{A\;\left(p_{2}-p_{1}\right)}$$

where *Q* is the volumetric flow rate (mol/s), *l* is the film thickness (m), *A* is the membrane area (m^2^) and $p_{1}$ and $p_{2}$, respectively, are the downstream and feed pressures.

An accepted unit of the permeability is 1 barrer unit, which equals the following:

$$1\;barrer=10^{-10}\times \frac{{\mathrm{cm}}_{\mathrm{STP}}^{3}\cdot \mathrm{cm}}{{\mathrm{cm}}^{2}\cdot s\cdot \mathrm{cmHg}}=3.35\times 10^{-16}\frac{\mathrm{mol}\cdot m}{m^{2}\cdot s\cdot \mathrm{Pa}}$$

where ${\mathrm{cm}}_{\mathrm{STP}}^{3}$ is the amount of gas in standard cubic centimeter.

Selectivity (α) is a crucial parameter to achieve high product purity and is defined as follows:

(2) $$\alpha =\frac{Y_{A}/Y_{B}}{X_{A}/X_{B}}$$

where Y and X are the molar fractions of the gases *A* and *B* in the permeate and the feed, respectively. An increase in the gas permeability of a membrane typically corresponds to a decrease in its selectivity and vice versa [7]. To obtain a high gas-diffusion selectivity in several gas pairs (O_2_/N_2_ and CO_2_/CH_4_), a size distribution of the elements based on the effective size of the molecules is required [31].

**Figure 1:**
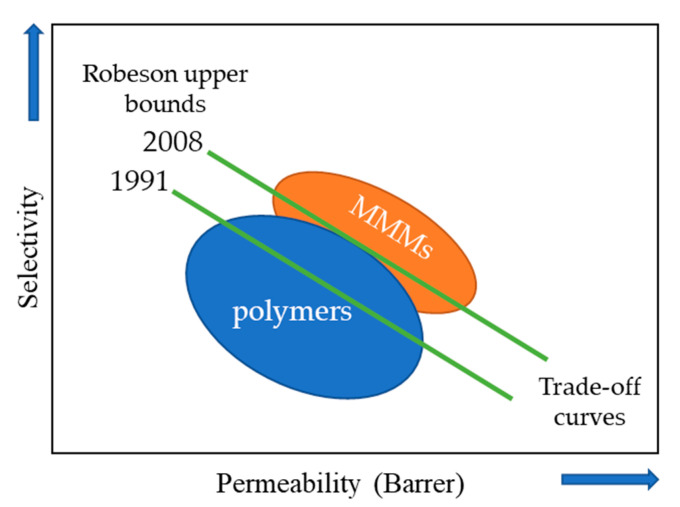
Diagram of the trade-off between selectivity and permeability.

The Robeson’s upper limit determines the permeability factor that membranes can achieve [32,33]. As an example of Robeson’s upper limit, a diagram is depicted in Figure 1, where a selectivity-versus-permeability plot is presented. In general, the separation performance of MMM is superior to that of organic polymers membranes [34].

Numerous researchers have explored inorganic membrane materials; some of them exhibited outstanding separation performance surpassing the Robeson’s upper-bound plots [35,36]. Ideally, the composite membranes should take advantage of both the filler and a rapid and selective gas transport [37,38]. The required characteristics to correctly choose the embedded phase typically contains a chemical adaptation for the dispersion in the polymeric matrix, a specific particle shape and morphology, and suitable properties on the overall transport performance.

Currently, the most studied membranes for gas separation are those with a polymeric base with additive fillers such as zeolites [39], metallic organic structures (MOF) [40], carbon molecular sieves [41,42], silica [43], carbon nanotubes (CNT) [44] and graphenes [45,46,47,48]. These membranes have been shown to overcome the permeability gradient that can combine the benefits of superior properties of gas transport and thermal resistance of molecular sieves with the mechanical properties and, also, good processing capacity [49,50,51].

An important key to obtain the best properties in MMM is the correct selection of the polymer materials and the fillers. In particular, this paper focuses on using CNT as fillers of MMM, since CNT are most attractive due to their superior properties compared to conventional ones and potentially provide a solution to the trade-off issues of the polymeric membrane. The present contribution intends to take into consideration different polymeric bases and mechanisms used in the separation of CO_2_ gas. Contributions from theoretical and experimental results found in the literature are discussed in order to understand the synergistic effect between CNT and MMM.

## 2. Mixed-Matrix Membranes with CNT as Fillers

**Figure 2:**
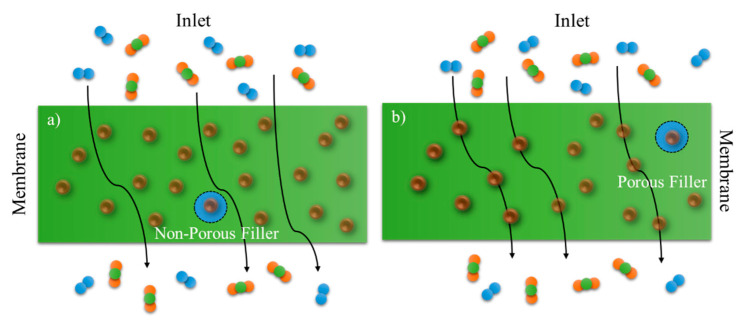
Transport mechanism with (**a**) conventional permeable particles and MMM (**b**) high aspect ratio.

A representation of the transport mechanism in MMMs, assuming a sieving effect of the inorganic phase, is depicted in Figure 2a. CO_2_/N_2_ separation is taken as a reference, but similar arguments can be made with other penetrant pairs. In Figure 2b, the separation performances are improved by increasing the so-called diffusivity–selectivity of the different penetrants in the membrane matrix. Well-defined microporous cavities in CNT offer high gas-diffusion coefficients, whereas their ultramicroporous apertures contribute to high diffusion selectivity. In this view, the geometry of the inorganic phase plays an important role. High-aspect-ratio structures can indeed lead to a better separation performance compared with conventional fillers. However, an ordered morphology for high-aspect-ratio nanoparticles is more difficult to achieve, and it can easily lead to defects and nonselective regions, thus decreasing the overall performance.

Polymers are normally used as membrane materials because of their low cost and ease of preparation. The diffusion of gas molecules through the polymeric matrix mainly depends on the characteristics of the free volume, such as the static cavities created by inefficient packaging of the chain and the transient spaces generated by the rearrangement of the thermally induced segment of the chain [52]. Researchers have significantly explored the different ways to change the polymer-chain packing to improve the performance of the membrane in terms of permeability and selectivity [53].

The diffusion coefficient in the polymeric membrane is related to the free volume available within the membrane matrix. In the case of porous membranes, the diffusivity of the molecules can be directly related to porosity (pore size and distribution) and tortuosity of pathways available for the molecules. A fine-tuning of these two characteristics can help in increasing the flux without affecting the membrane selectivity [54,55]. On the other side, in nonporous membranes, the free volume represents the space between the polymeric chains available for the permeation in the membrane matrix [56,57]. For this reason, over the last decade, polymer research has focused on the development of high free volume polymers that are capable of achieving large transmembrane fluxes.

At the beginning of the 21st century, the synthesis of new and attractive polymeric materials (e.g., polymers of intrinsic microporosity (PIM) and thermally rearranged polymers), together with the development of new nano-sized particles, opened up a completely new field for hybrid membranes. Fillers such as MOFs, zeolite imidazolium frameworks (ZIF), CNT and other spherical and layered nanostructures were dispersed in several polymeric materials, showing promising results in gas separation [58,59].

Therefore, mixed-matrix membranes (MMMs) are heterogeneous membranes which consist of a polymer based MOF with additive fillers and can be developed in different shapes, such as flat sheets and hollow-fibers. Inorganic materials, which are used as the dispersed phase in MMMs, have a unique structure, surface chemistry, and mechanical strength [60]. Among different fillers, CNTs have good separation and mechanical properties for applications of gas-separation processes [61]. These fillers have been widely implemented because of their conductivity; their affinity to small molecules, such as CO_2_, CH_4_ and others gases; and because their ability to endure high temperatures and aggressive chemicals [62].

The use of fillers in the membrane matrix can improve the selective features of highly permeable polymers and consequently have different effects on the transport properties; they can also increase the stability of the membrane layers operating under high-pressure conditions [63]. There is a research trend related to MMMs to obtain high gas-separation efficiencies with new support materials and fillers. To select the polymeric matrix for the MMMs, it is necessary to know the main physicochemical factors that influence permeability, such as mobility of polymer chains, intersegmental spacing and interactions of the polymer with the penetrating gas [64,65,66,67].

Fillers are classified according to their morphology and transport mechanisms, such as zero dimensional (0D), one dimensional (1D) and two dimensional (2D), these nanofillers participate in the packaging of the polymer chain and improve the free volume characteristics [68]. One-dimensional fillers are renowned in nanocomposites for enhancing structural stability of the polymeric matrices due to their high aspect ratio (length-to-diameter ratio) [69,70,71].

**Figure 3:**
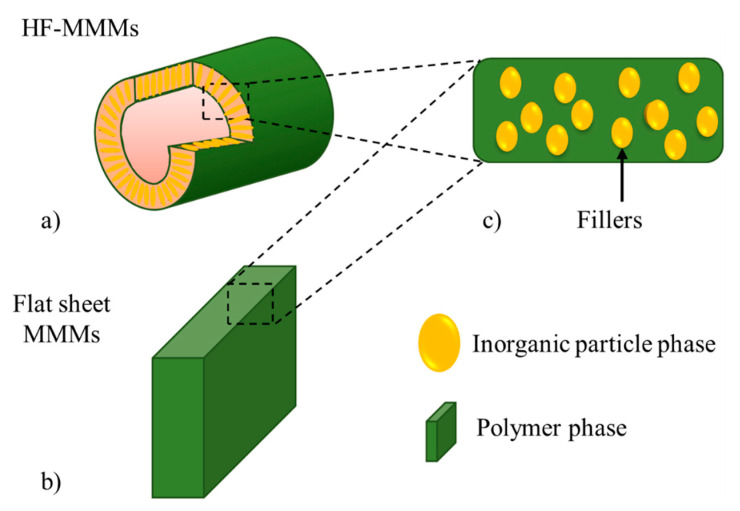
MMM with fillers (**a**) asymmetrical hollow fiber or (**b**) flat sheets. In (**c**), the fillers are homogeneously distributed in the polymer phase.

Fillers dispersed at a nanometer level in a polymer matrix have been identified to potentially provide a solution to the trade-off issues of the polymeric membrane, as well as solving the inherent brittleness problems found in polymeric membranes [72,73,74]. The polymer and filler determine the morphology and performance of the MMMs. Figure 3 shows two configurations of MMMs with fillers. These membranes can be composed of symmetric and flat sheets with fillers or of hollow-fiber with a dense skin with fillers in an asymmetric arrangement.

The incorporation of CNT in polymeric membranes has received attention for the development of new gas-separation membrane technologies, such as MMMs [75]. CNT fillers have an affinity for CO_2_ absorption and transport capabilities contributing to a greater interest in membranes for gas separation [76]. CNTs are 1D nanomaterials with excellent mechanical and thermal properties and, generally, are classified in function of the number of walls, into single-walled carbon nanotubes (SWCNTs) and multi-walled nanotubes (MWCNTs). The increased use of CNTs in membrane applications can be attributed to unique separation properties, such as their surface area and ability to improve mechanical strength with small filler content and a good control of pore size at the nanoscale [77,78].

SWCNTs consist of a rolled sheet of graphite that has a cylindrical shape with a diameter of up to 1.5 nm. They possess a high surface area with interstitial channels, while providing an increase of adsorption sites with high binding energy. On the other hand, MWCNTs consist of two or more concentrically formed cylinders with a distance of 0.35 nm, similar to the basal plane separation in graphite. They can have lengths of tens of microns and diameters between 2 and 100 nm [79]. They contain interlayer spaces that might act as adsorption sites for smaller molecules [80]; in general, the application of CNT improves the gas-separation behavior of MMM.

To improve the distribution of CNT as fillers in MMMs, different dispersion techniques adding polar groups to the CNT side walls have been explored, such as direct suspension in polymer solution by sonication or by surface oxidation, and insertion of hydrophilic functional groups on the surface to disperse them uniformly in the polymer matrix and to improve adhesion [81,82]. Covalent and non-covalent functionalization is used to improve the dispersion of CNTs in polymeric matrices [83]. The covalent functionalization is based on the bond between carbon and other functional groups; it can take place in the defects and at the end of the caps or in the side walls, improving the solubility in solvents with different chemical structures [84]. The non-covalent functionalization improves the dispersion of CNTs by the adsorption of functional groups on the nanotube surface, making them soluble in organic and aqueous solvents; additionally, it does not damage the side walls and, therefore, it does not affect the final properties of the material [85].

## 3. Application of CNT–MMM in CO_2_ Separation

The nanotubes have a vast range of applications due to their inherent properties that can improve the own chemistry, mechanical and electrical properties [86]. In this section, MMM applications for CO_2_ separation, with a focus on the influence of the addition of functionalized and no-functionalized CNT to MMM, are depicted, and, also, some results about the combination of CNT and other compounds added to MMM are discussed.

### 3.1. Influence of CNT as Fillers in MMM

Several researchers have studied the influence of CNT to improve MMM, in general they found that CO_2_ permeability rises with an increase in the CNT content, which improves, the mechanical strength without impairing the gas-separation performance. For instance, in Reference [87] researchers found better results by adding to a brominated poly (2,6-diphenyl-1,4-phenylene oxide) (BPPOdp) membrane 9 wt% of SWCNT and 5 wt% of MWCNT. Another investigation [88] demonstrated a better permeability by adding only 2 wt% of MWNT to a N-methyl-2-pyrrolidone membrane (at higher quantities of MWNT the membranes deteriorates due to the formation of agglomerates); the authors attributed this performance to the effective dispersion of the nanotubes in the polymer. The addition of carbon nanotubes to the polyimide membranes matrices improves their properties due to the uniform dispersion achievement of the nanotubes in the polymer matrix, which could play the role of a polymeric-chain crosslinker.

Furthermore, MWCNTs accelerate the transport of easily condensable gases, such as CO_2_ and O_2_, which increase the gas permeability by diminishing the gas diffusion resistance; for example, results obtained in Reference [89] demonstrate a separation factor of CO_2_/N_2_ of 2 to 4 times higher than that of pure carbon membrane prepared under same procedure and experimental conditions. The CNT-enhanced gas permeability could be attributed to the increase in gas diffusivity, which came from the increase in free volume and gas transportation inside the nanotubes [90].

### 3.2. Influence of Functionalized CNT as Fillers in MMM

The functionalization of CNTs was studied in order to improve their deposition in the polymeric matrix, and to improve their efficiency, several functional groups were tested, such as hydroxyl (•OH), carboxylic (-COOH), amino (•NH_2_) and isocyanate (-NCO) groups.

The influence of functionalized MWCNT in MMMs based on PC fabrication was studied in Reference [91]; the results show that functionalized nanotubes provide better performance and also an increase of CO_2_/N_2_ selectivity. Moreover, the authors have observed that an increase in feed pressure resulted in an acceptable improvement in permeability and selectivity of test gases. Additional studies of the influence of pressure and of temperature on the performance of MMM with functionalized MWCNT mixed with Triton X100 was reported in Reference [92]. They found that the gas-separation performance of the prepared membranes remarkably depends on the properties of permeating gas molecules and the operating pressure and temperature; by slightly increasing those factors to 3 bar and 30 °C, they obtained a better performance in gas separation, surpassing the Robeson’s upper bound for CO_2_/N_2_ separation with 4 wt% of MWCNT.

Concerning the influence of amino group in MWCNT–MMM, Reference [93] demonstrated the limited effect of the •NH_2_ group on gas selectivity; nevertheless, they have observed a remarkably increase (almost 3 times) on CO_2_ permeability when 25 wt% of functionalized CNT were used in MMM. Similar results were obtained by Reference [94], where the •NH_2_ group showed its positive impact on CO_2_ permeability over pristine nanotubes or even over nanotubes functionalized with •OH groups.

Carboxylic and hydroxyl groups have a strong interaction with CO_2_, they increase the solubility coefficient of polar gases and also the CO_2_ permeability coefficient, as was demonstrated in Reference [95]. Results obtained herein showed that the functionalized MWCNTs, added in 3 wt% to MMM, were cut into short ropes containing •OH and -COOH functional groups on the surface of MWCNTs. The influence of -COOH functional groups were also demonstrated in Reference [96], where functionalized SWNT–MMM exhibited the best separation performance at 2 wt% filler content, which can be attributed to the improved interfacial affinity with carboxylic acid functionalized surface of SWNTs. In this case, the remarkably improved performance could be attributed to the CNT with smaller aspect ratio that had more open ends for gas molecules to pass through, as well as functionalized surface of carbon nanotubes improving the solubility selectivity and finally to the higher purity of the modified carbon nanotubes as fillers.

The functional group -NCO on MWCNTs could be used to overcome the filler aggregation in the membrane and to increase gas permeation and CO_2_/CH_4_ and CO_2_/N_2_ gas selectivity, as confirmed in Reference [97]. The high performance of the MWCNT–NCO membrane could be explained because of the high polarity of -NCO and better compatibility with polymer chains of MMM (i.e., polyurethane). Additional results that support such data were reported in Reference [98]; their results demonstrated that using 0.3 wt% loading MWCNT–NCO had the best performance over nanotubes functionalized with -COOH and -NH_2_ and, additionally, significant CO_2_/N_2_ and CO_2_/CH_4_ exceeding Robeson’s upper limit. In summary, MWCNT–NCO have high polarity and better interaction with polymer chains, as well as better physicochemical properties and a higher gas-separation performance.

Techniques of synthesis of MMM also have an influence on membrane characteristics, here, are analyzed only those who exceeded Robeson’s upper limit. In particular, results reported in References [90,98,99] used PEBAX-1657 as a polymeric base, comprising 40 wt% Nylon 6 PA6 segment and 60 wt% polyethylene oxide (PEO) segment. This is a thermoplastic elastomer that combines linear polymer chains that provide mechanical strength with flexible polymer interlayers. Pebax-1657 copolymers showed particularly high selectivity for polarizable/non-polar gas pairs, such as CO_2_/N_2_, making it a better choice than other polymers as a polymeric matrix for MMMs.

To obtain the desirable attributes of a single composite, (i.e., flexibility, processability, high selectivity for polarizable/non-polar gas separation, and the mechanical strength and thermal stability of CNT) of Pebax-1657, the authors of results reported in References [92,97,100] used different techniques, such as solution casting method and solvent evaporation method, where they disperse the functionalized MWCNTs in solvents and then mix them with the polymeric base by ultrasonic technique. For example, Reference [101] prepared MMMs by using the solution-casting method and solvent evaporation method. Pebax pellets (0.8 g) were dissolved in the ethanol-and-water mixed solvent (12 mL, mass ratio of 7:3), with stirring at 80 °C for 4 h. The MWCNTs–ZIF-8 were added into another ethanol-and-water mixed solvent (11.5 mL, mass ratio of 7:3) with ultra-sonication and stirring. Then the fillers solution was added into the Pebax solution to get a uniform mixed solution by stirring and ultra-sonicate. Subsequently, the mixed solution was poured into a plastic Petri dish and dried at room temperature for 24 h. Finally, the prepared membranes were dried again at 25 °C, in a vacuum oven, to remove the solvent. The functionalized MWCNTs improved the compatibility between Pebax and ZIF-8, which contributed to the improvement of CO_2_/N_2_ selectivity of MMMs. The insertion structure was the key to solve the aggregation problems of ZIF-8 particles, which could improve the dispersion of ZIF-8 particles. It is found that the MWCNTs could be used as a bridge of rapid transmission for CO_2_ molecules, contributing to the improvement of CO_2_ permeability of MMMs.

This type of MMMs-making process was similarly used by References [98,102]; their variables were the concentration of the CNTs and functional groups (MWCNT–COOH or MWCNT–NCO), which lead to good interfacial adhesion, allowing higher dispersion and obtaining a high efficiency to overcome the Robenson’s upper limit. Additional results with MMMs elaborated by the solution casting method with a PU membrane and different amounts of MWCNTs (with functionalized groups -COOH and -NCO) have shown that the presence of MWCNT–NCO led to an increase in CO_2_/CH_4_ and CO_2_/N_2_ selectivity up to 14% and 51%, respectively, due to the high polarity of -NCO and its improved compatibility with polymer chains [97].

Another example that exceeded Robenson’s upper limit was reported in Reference [92], where they combined different fillers. The high performance of the membrane was due to the high polarity of -NCO and better compatibility with polymer chains with Triton X-100 to modify MWCNTs. Two-layer Pebax/PES mixed-matrix membranes containing CNT–COOH, CNT–NH_2_ and CNT–X100 as fillers were prepared via solvent-evaporation phase-separation technique. For this purpose, a 12 wt% Pebax solution in formic acid was prepared by dissolving the polymer in the solvent, and then a certain amount of the filler samples was dispersed into the polymeric solution. To avoid agglomeration of the MWCNTs, the obtained solution was periodically stirred for 15 min and sonicated for 30 min at least 3 h. The gas-separation performance of the prepared membranes was found to be significantly dependent on the properties of the permeant gas molecules and the operating pressure and temperature. In the case of CO_2_/N_2_ separation, the membrane containing 4 wt% CNT–X100 filler exceeded the Robeson upper limit and exhibited the highest gas-separation performance.

### 3.3. Influence of CNT and Other Compounds Added to MMM

In order to increase the performance of MMMs with CNTs as fillers, additional compounds could be mixed with CNTs; some of these materials could be graphene oxides (GO), zeolites or PIM). Additional supports have been studied, such as hydrogel (i.e., N-isopropylacrylamide hydrogel, NIPAM) or the addition of SiO_2_ nanoparticles in liquid-like hybrid materials.

Incorporation of similar quantities (i.e., 1:1 wt%) of CNT and GO into an MMM was studied in Reference [103]. Results showed that MMMs containing CNTs had higher CO_2_ permeability but lower selectivity. Meanwhile, MMMs containing GO nanosheets had higher selectivity but lower permeability. MMMs using both CNTs and GO as fillers had both improved CO_2_ permeability and CO_2_/CH_4_ and CO_2_/N_2_ selectivity over pure polymer membrane. The addition of GO nanosheet also improve the dispersion of CNT in a membrane as described in Reference [100]. It was observed that the nanomaterials properties, such as dispersion and hydrophilicity, could affect the reactivity of interfacial polymerization, which, in turn, altered the characteristics of selective layer. Results showed that the incorporation of GO and CNT modified with amino acids improved CO_2_ permeability and CO_2_/N_2_. Moreover, this study demonstrated that GO nanosheets serve as good dispersants for nanotubes in aqueous solution and prevent the latter from aggregating attributed to the GO high dispersion. Deposition of the nanotubes onto the basal plane of GO also reduced the nanotube agglomeration tendency. Synergy between the nanotubes and GO combinations boosted both the permeance and selectivity of the thin-film nanocomposite membrane.

Relating to the use of zeolites to increase MWCNT–MMM performance, some studies showed high separation performance of CO_2_ [101]; these results were confirmed in an additional study involving CO_2_ removal from post-combustion flue gas. An additional advantage of using zeolites is the fact that MMMs can operate in wet conditions compared to usually dry conditions having, at the same time, selectivity above Robeson’s upper limit [104].

The intrinsic porosity of some polymers with functionalized MWCNTs has been studied, and good results on CO_2_ separation performance were attained, as detailed in Reference [102]. This work reported the structure of PIM, as well as the effect of nanofillers obtaining high CO_2_ permeability with a desirable CO_2_/N_2_ separation factor with 7.5 wt% of functionalized MWCNTs, placed on the special microporous structure of the PIM. The functionalized carbon nanotubes had a uniform dispersion owing to the presence of functional groups, such as the hydroxyl groups, carboxyl groups and amino group.

Finally, interesting research of new phases, such as hydrogels to enhance MMM efficiency, is detailed in Reference [105]; here the researchers showed results of MMMs fabricated by incorporating NIPAM-CNTs composite filler into poly (ether-block-amide) (Pebax-1657) matrix for efficient CO_2_ separation. MMM containing 5 wt% NIPAM-CNTs exhibited the highest CO_2_ permeability and high CO_2_/CH_4_ and CO_2_/N_2_ selectivity, transcending 2008 Robeson upper bound line. The extraordinary smooth one-dimensional nanochannels of CNTs and super water hygroscopicity of NIPAM hydrogels are key factors contributing the efficient CO_2_ separation in MMM.

A mixture of CNT with SiO_2_ nanoparticles under a liquid-like phase incorporated into a Pebax-1657 matrix for CO_2_ capture was studied by Reference [99]. Specifically, the prepared membrane presented optimal performance regarding CO_2_ permeability and CO_2_/N_2_ separation factor, whereas improvements in CO_2_ permeability are obtained by overcoming the Robeson upper bound. In this study, the liquid-like CNT/SiO_2_ nanoparticles contributed to the formation of good interfacial compatibility, owing to their good dispersion properties, which significantly improved the gas permeability.

A comparison of permeability and selectivity is summarized in Table 1, where the appearing values are those obtained by the works of the authors previously described. This table also specifies the membrane type and the CNT quantities utilized under different temperature and pressure conditions.

**Figure 4:**
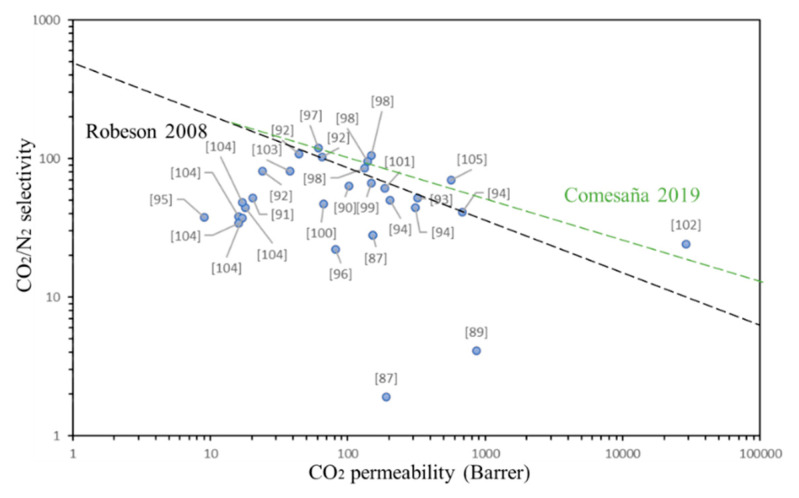
Upper bound correlation for CO_2_/N_2_ separation.

**Figure 5:**
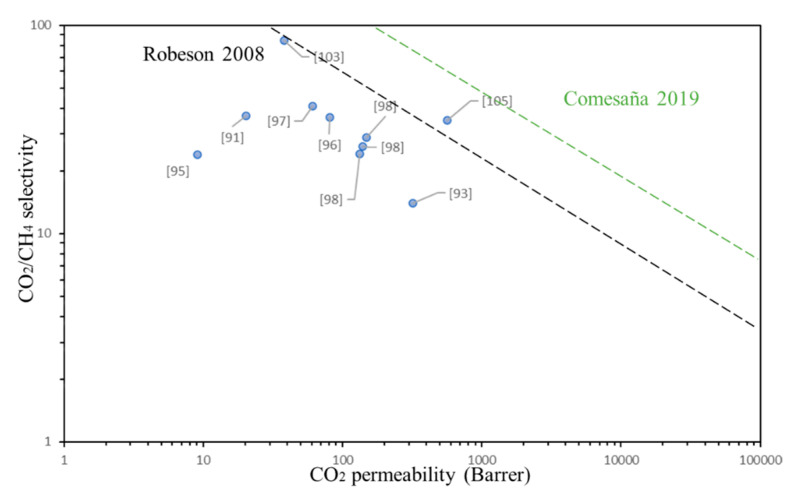
Upper bound correlation for CO_2_/CH_4_ separation.

Some key data given in Table 1, related to the upper-bound data for CO_2_/N_2_ and CO_2_/CH_4_ separation, are respectively plotted in Figure 4 and Figure 5. Although a large amount of data has been generated since 2007, a minor shift in the upper-bound relationship is noted; membrane separation performances are still marginal, with the CO_2_/CH_4_ separation being more difficult to be applied on industrial sector. The ladder polymers, PEBAX, BTDA–TDI/MDI (P84) co-polyimide, PEBA, polyurethane (PU) and composites with hydrogel, comprise points on the upper bound as with the two gas pairs noted above.

Until now the Robeson‘s upper limit has been largely used as a reference in several papers; in order to make more attractive the separation membranes (i.e., energy and cost efficiencies), newer parametric estimates were reported in Reference [106]. This upper limit was also added to Figure 4 and Figure 5 as Comesaña 2019 (green dotted line). As can be seen from these figures the CNT–MMM used to separate CO_2_/CH_4_ are still far from their industrial application and, concerning the CO_2_/N_2_ separation, PEBA polymeric matrix, Cardo-PIM-1 with functionalized CNT and CNT–MMM with hydrogel, seems to have better possibilities to be scaled-up. In fact, this last one membrane with hydrogel [105] seems to have higher possibilities to be enhanced and be applied in both, separation of CO_2_/CH_4_ and CO_2_/N_2_.

From the results discussed above, it was found that the gas-separation performance of the prepared membranes remarkably depends on the properties of permeating gas molecules and the operating pressure and temperature. The efficiency of the membranes was improved by adding functionalized CNTs as membrane fillers and additional compounds, such as zeolites or hydrogel. The results also revealed that the uniform CNT dispersion lead to enhanced gas permeation properties; however, using high concentration of CNTs results in the deterioration of the final properties of the membranes due to the agglomerates formation. So, one challenge to improve the MMM performance is to uniformly distribute the functionalized CNT mixed with other compounds (e.g., zeolites and MOF) in liquid and hydrogel surfaces. The permeation mechanism is the result of the cooperativity of the solution–diffusion mechanism of the gas through the polymeric matrix and the gas diffusion mechanism through the formatted polymeric free volume and/or filler–polymer interfaces. Concerning the CNT functionalization, the -NCO group created showed better physicochemical properties and exhibited a high gas-separation performance; its high polarity and better compatibility with polymer chains can improve the permeability of CO_2_/N_2_.

## 4. Conclusions

From the review of these results, it is clearly established that the MMM efficiency can be improved by adding CNT as fillers of those membranes and, even better results can be obtained if CNT are functionalized and if the additives (GO, zeolites, hydrogel and liquid-like SiO_2_ nanoparticles) are used. These conditions allow for an increased fillers dispersion and stabilization in the matrix, which in turn, a better performance of CNT–MMM in terms of permeability without sacrificing selectivity can be achieved.

Future research on CNT–MMM should be focused on diminishing the quantity of CNT or any nanostructure in order to reduce the cost of membranes, enhancing then, their performance. Research should also focus on obtaining key functional groups with green methods and thus, for instance, avoiding strong acids. Finally, studies have to be enriched under real conditions considering for example, higher fluxes, temperatures and relative humidity, characteristic of natural and gas power plants, in order to determine the lifetime of the CNT–MMM and their performance.

## Figures and Tables

**Table 1:** Permeability and selectivity values of different CNT–MMM used in gas-separation processes.

Fillers	Polymer Matrix	CNT wt%	Pressure (Bar)	Temperature (°C)	Permeability (Barrer) CO_2_	Selectivity (CO_2_/N_2_)	Selectivity (CO_2_/CH_4_)	Reference
CNTs	Brominated poly(2,6-diphenyl-1,4-phenylene oxide)	5	0.689	25	153	28	-	[87]
CNTs	Polyimide	15	1	25	866.6	4.1	-	[89]
C-MWCNTs	PC/PEG	10	2	25	20.32	52.10	36.64	[91]
SWNT	PEBA MMMs	5	2.3	21	102	63	-	[90]
MWNTs–NH2	Pebax 1657	23	7	34.85	320	52	14	[93]
CNTs-GO	Matrimids	5	4 × 10^−5^	25	38.07	81	84.6	[103]
CNTs	Pebax		2	25	567	70	35	[105]
MWCNTs-COOH-4	Pebax	4	3	30	24	81	-	[92]
MWCNTs-X100	Pebax	4	3	30	65	103	-	[92]
MWCNTs-NH_2_	Pebax	4	3	30	44	108	-	[92]
MWNT-COOH-OH	polyimide (PI)	3	1	15	9.06	37.74	24	[95]
MWNT	Pebax	5	7	34.85	202	50	-	[94]
MWNT	Pebax	10	7	34.85	310	44	-	[94]
MWNT	Pebax	15	7	34.85	680	41	-	[94]
CNTs	Brominated poly(2,6-diphenyl-1,4-phenylene oxide)	5	0.689	25	153	28	-	[87]
CNT/ZIF-301(6)	PSF	10	2	25	16	38	-	[104]
CNTs/ZIF-301(12)	PSF	8	2	25	17	37	-	[104]
CNTs/ZIF-301(18)	PSF	6	2	25	18	44	-	[104]
CNTs/ZIF-301(24)	PSF	4	2	25	17	48	-	[104]
CNTs/ZIF-301(30)	PSF	2	2	25	16	34	-	[104]
CNTs–GO	TFN	Ratio CNT and GO 1:1	4	70	66.3	47.1	-	[100]
MWCNT–COOH	PEBA	0.75	10	25	132.30	85.32	24.18	[98]
MWCNT–NCO	PEBA	0.3	10	25	148.86	104.92	28.95	[98]
MWCNT–NH_2_	PEBA	0.5	10	25	139.53	95.62	26.28	[98]
CNT/SiO_2_ (NOHM)	Pebax-1657	10	20	66.5	148.3	66.5	-	[99]
MWCNTs	BTDA–TDI/MDI (P84) co-polyimide	2	1	25	190.5	1.9	-	[87]
MWCNTs ZIF-8–8	Pebax1657	8	5	35	186.3	61.3	-	[101]
MWCNT–NCO	polyurethane (PU)	0.3	10	30	61.36	119.51	40.87	[97]
f-MWCNTs	bis(phenyl) fluorene-based PIMs (Cardo-PIM-1)	7.5	1	25	2.9 × 10^4^	24.2	-	[102]
AP-SWNTs	6FDA-TP polyimide	2	16.4	35	81	22	36	[96]

## Data Availability

Not applicable.

## References

[1] Zou C., Li Q., Hua Y., Zhou B., Duan J., Jingui D. (2017). Mechanical Synthesis of COF Nanosheet Cluster and Its Mixed Matrix Membrane for Efficient CO2 Removal. ACS Appl. Mater. Interfaces.

[2] Zhang Y., Wang H., Liu J., Hou J., Zhang Y. (2017). Enzyme-embedded metal-organic framework membranes on polymeric substrates for efficient CO2 capture. J. Mater. Chem. A.

[3] World Meteorological Organization (WMO) (2015). WMO Greenhouse Gas Bulletin: The State of Greenhouse Gases in the Atmosphere Based on Global Observations Through.

[4] Zhang Z., Chen F., Rezakazemi M., Zhang W., Lu C., Chang H., Quan X. (2018). Modeling of a CO_2_-piperazine-membrane absorption system. Chem. Eng. Res. Des..

[5] De Vos R.M. (1998). High-Selectivity, High-Flux Silica Membranes for Gas Separation. Science.

[6] Shiflett M.B. (1999). Ultrasonic Deposition of High-Selectivity Nanoporous Carbon Membranes. Science.

[7] Merkel T.C., Freeman B.D., Spontak R.J., He Z., Pinnau I., Meakin P., Hill A.J. (2002). Ultrapermeable, Reverse-Selective Nanocomposite Membranes. Science.

[8] Baker R.W., Low B.T. (2014). Gas Separation Membrane Materials: A Perspective. Macromolecules.

[9] Sazali N., Salleh W.N.W., Ismail A.F., Wong K.C., Iwamoto Y. (2018). Exploiting pyrolysis protocols on BTDA-TDI/MDI (P84) polyimide/nanocrystalline cellulose carbon membrane for gas separations. J. Appl. Polym. Sci..

[10] Basile A., Gallucci F. (2011). Membranes for Membrane Reactors: Preparation, Optimization and Selection.

[11] Nandi B., Uppaluri R., Purkait M. (2008). Preparation and characterization of low cost ceramic membranes for micro-filtration applications. Appl. Clay Sci..

[12] Perry R.H., Green D. (2007). Perry’s Chemical Engineers’ Handbook.

[13] Gandia L., Arzamedi G., Dieguez P. (2013). Renewable Hydrogen Technologies: Production, Purification, Storage, Applications and Safety.

[14] Venna S.R., Lartey M., Li T., Spore A., Kumar S., Nulwala H.B., Luebke D.R., Rosi N.L., Albenze E. (2015). Fabrication of MMMs with improved gas separation properties using externally-functionalized MOF particles. J. Mater. Chem. A.

[15] Pal R. (2008). Permeation models for mixed matrix membranes. J. Colloid Interface Sci..

[16] Widjojo N., Chung N.T.-S., Kulprathipanja S. (2008). The fabrication of hollow fiber membranes with double-layer mixed-matrix materials for gas separation. J. Membr. Sci..

[17] Amooghin A.E., Mashhadikhan S., Sanaeepur H., Moghadassi A., Matsuura T., Ramakrishna S. (2019). Substantial breakthroughs on function-led design of advanced materials used in mixed matrix membranes (MMMs): A new horizon for efficient CO_2_ separation. Prog. Mater. Sci..

[18] Japip S., Xiao Y., Chung T.-S. (2016). Particle-Size Effects on Gas Transport Properties of 6FDA-Durene/ZIF-71 Mixed Matrix Membranes. Ind. Eng. Chem. Res..

[19] Basu S., Odena A., Vankelecom I.F. (2011). MOF-containing mixed-matrix membranes for CO_2_/CH_4_ and CO_2_/N_2_ binary gas mixture separations. Sep. Purif. Technol..

[20] Abetz V., Brinkmann T., Dijkstra M., Ebert K., Fritsch D., Ohlrogge K., Paul D., Peinemann K.-V., Pereira-Nunes S., Scharnagl N. (2006). Developments in Membrane Research: From Material via Process Design to Industrial Application. Adv. Eng. Mater..

[21] Soltani B., Asghari M. (2017). Effects of ZnO Nanoparticle on the Gas Separation Performance of Polyurethane Mixed Matrix Membrane. Membranes.

[22] Rafiq S., Deng L., Hägg M.-B. (2016). Role of Facilitated Transport Membranes and Composite Membranes for Efficient CO_2_ Capture—A Review. ChemBioEng Rev..

[23] Wee S.-L., Tye C.-T., Bhatia S. (2008). Membrane separation process—Pervaporation through zeolite membrane. Sep. Purif. Technol..

[24] Lozano L., Godínez C., Ríos A.D.L., Hernández-Fernández F., Sánchez-Segado S., Alguacil F.J. (2011). Recent advances in supported ionic liquid membrane technology. J. Membr. Sci..

[25] Korelskiy D., Ye P., Fouladvand S., Karimi S., Sjöberg E., Hedlund J. (2015). Efficient ceramic zeolite membranes for CO_2_/H_2_ separation. J. Mater. Chem. A.

[26] Kumbhare M.B., Sapkal V.S., Sapkal R.S. (2018). A Review: Membrane for CO_2_ Separation from Syngas and Hydrogen. Int. J. Basic Appl. Res..

[27] Baker R.W. (2002). Future Directions of Membrane Gas Separation Technology. Ind. Eng. Chem. Res..

[28] Swaidan R., Ma X., Litwiller E., Pinnau I. (2013). High pressure pure- and mixed-gas separation of CO_2_/CH_4_ by thermally-rearranged and carbon molecular sieve membranes derived from a polyimide of intrinsic microporosity. J. Membr. Sci..

[29] Shantarovich V.P., Kevdina I.B., Yampolskii Y.P., Alentiev A.Y. (2000). Positron Annihilation Lifetime Study of High and Low Free Volume Glassy Polymers: Effects of Free Volume Sizes on the Permeability and Permselectivity. Macromolecules.

[30] Raj K.R., Sunarti A.R. (2019). Preliminary Fractional Factorial Design (FFD) study using incorporation of Graphene Oxide in PVC in mixed matrix membrane to enhance CO_2_/CH_4_ separation. IOP Conf. Ser. Mater. Sci. Eng..

[31] Shen Y., Lua A.C. (2012). Preparation and characterization of mixed matrix membranes based on PVDF and three inorganic fillers (fumed nonporous silica, zeolite 4A and mesoporous MCM-41) for gas separation. Chem. Eng. J..

[32] Robeson L.M. (1991). Correlation of separation factor versus permeability for polymeric membranes. J. Membr. Sci..

[33] Robeson L.M. (2008). The upper bound revisited. J. Membr. Sci..

[34] Hunger K., Schmeling N., Jeazet H.B.T., Janiak C., Staudt C., Kleinermanns K. (2012). Investigation of Cross-Linked and Additive Containing Polymer Materials for Membranes with Improved Performance in Pervaporation and Gas Separation. Membranes.

[35] Adams R.T., Lee J.S., Bae T.-H., Ward J.K., Johnson J., Jones C.W., Nair S., Koros W.J. (2011). CO_2_–CH_4_ permeation in high zeolite 4A loading mixed matrix membranes. J. Membr. Sci..

[36] Dong G., Li H., Chen V. (2013). Challenges and opportunities for mixed-matrix membranes for gas separation. J. Mater. Chem. A.

[37] Wang M., Wang Z., Li N., Liao J., Zhao S., Wang J., Wang S. (2015). Relationship between polymer–filler interfaces in separation layers and gas transport properties of mixed matrix composite membranes. J. Membr. Sci..

[38] Dechnik J., Gascon J., Doonan C.J., Janiak C., Sumby C.J. (2017). Mixed-Matrix Membranes. Angew. Chem. Int. Ed..

[39] Barankova E., Pradeep N., Peinemann K.-V. (2013). Zeolite-imidazolate framework (ZIF-8) membrane synthesis on a mixed-matrix substrate. Chem. Commun..

[40] Arjmandi M., Pakizeh M. (2014). Mixed matrix membranes incorporated with cubic-MOF-5 for improved polyetherimide gas separation membranes: Theory and experiment. J. Ind. Eng. Chem..

[41] Vu D.Q., Koros W.J., Miller S.J. (2003). Mixed matrix membranes using carbon molecular sieves: I. Preparation and experimental results. J. Membr. Sci..

[42] Koresh J.E., Soffer A. (1987). The Carbon Molecular Sieve Membranes. General Properties and the Permeability of CH_4_/H_2_ Mixture. Sep. Sci. Technol..

[43] Zornoza B., Téllez C., Coronas J. (2011). Mixed matrix membranes comprising glassy polymers and dispersed mesoporous silica spheres for gas separation. J. Membr. Sci..

[44] Zhao Y., Jung B.T., Ansaloni L., Ho W.W. (2014). Multiwalled carbon nanotube mixed matrix membranes containing amines for high pressure CO_2_/H_2_ separation. J. Membr. Sci..

[45] Mahmoudi E., Teow Y.H., Mohammad A.W., Ba-Abbad M.M. (2020). The Effect of Graphene Oxide (GO) Loading for the Enhancement of Nylon 6,6-GO Mixed-matrix. Membr. Perform..

[46] Zhu W., Qin Y., Wang Z., Zhang J., Guo R., Li X. (2019). Incorporating the magnetic alignment of GO composites into Pebax matrix for gas separation. J. Energy Chem..

[47] Li X., Cheng Y., Zhang H., Wang S., Jiang Z., Guo R., Wu H. (2015). Efficient CO2 Capture by Functionalized Graphene Oxide Nanosheets as Fillers To Fabricate Multi-Permselective Mixed Matrix Membranes. ACS Appl. Mater. Interfaces.

[48] Quan S., Li S.W., Xiao Y.C., Shao L. (2017). CO 2 -selective mixed matrix membranes (MMMs) containing graphene oxide (GO) for enhancing sustainable CO 2 capture. Int. J. Greenh. Gas Control..

[49] Goh P., Ismail A., Sanip S., Ng B., Aziz M. (2011). Recent advances of inorganic fillers in mixed matrix membrane for gas separation. Sep. Purif. Technol..

[50] Wijenayake S.N., Panapitiya N.P., Versteeg S.H., Nguyen C.N., Goel S., Balkus J.K.J., Musselman I.H., Ferraris J.P. (2013). Surface Cross-Linking of ZIF-8/Polyimide Mixed Matrix Membranes (MMMs) for Gas Separation. Ind. Eng. Chem. Res..

[51] Ismail A., Rahim N., Mustafa A., Matsuura T., Ng B., Abdullah S., Hashemifard S.A. (2011). Gas separation performance of polyethersulfone/multi-walled carbon nanotubes mixed matrix membranes. Sep. Purif. Technol..

[52] Hamid M.R.A., Jeong H.-K. (2018). Recent advances on mixed-matrix membranes for gas separation: Opportunities and engineering challenges. Korean J. Chem. Eng..

[53] Ismail A., Goh P., Sanip S., Aziz M. (2009). Transport and separation properties of carbon nanotube-mixed matrix membrane. Sep. Purif. Technol..

[54] Krishna R., van Baten J.M. (2011). Maxwell–Stefan modeling of slowing-down effects in mixed gas permeation across porous membranes. J. Membr. Sci..

[55] Saito Y., Hirai K., Emori H., Murata S., Uetani Y., Kii K. (2004). Carrier Diffusivity in Porous Membranes. J. Phys. Chem. B.

[56] Nuhnen A., Dietrich D., Millan S., Janiak C. (2018). Role of Filler Porosity and Filler/Polymer Interface Volume in Metal–Organic Framework/Polymer Mixed-Matrix Membranes for Gas Separation. ACS Appl. Mater. Interfaces.

[57] Kudo Y., Mikami H., Tanaka M., Isaji T., Odaka K., Yamato M., Kawakami H. (2020). Mixed matrix membranes comprising a polymer of intrinsic microporosity loaded with surface-modified non-porous pearl-necklace nanoparticles. J. Membr. Sci..

[58] Rezakazemi M., Amooghin A.E., Montazer-Rahmati M.M., Ismail A.F., Matsuura T. (2014). State-of-the-art membrane based CO_2_ separation using mixed matrix membranes (MMMs): An overview on current status and future directions. Prog. Polym. Sci..

[59] Noble R.D. (2011). Perspectives on mixed matrix membranes. J. Membr. Sci..

[60] Kiadehi A.D., Rahimpour A., Jahanshahi M., Ghoreyshi A.A. (2015). Novel carbon nano-fibers (CNF)/polysulfone (PSf) mixed matrix membranes for gas separation. J. Ind. Eng. Chem..

[61] Nour M., Berean K., Balendhran S., Ou J.Z., Du Plessis J., McSweeney C., Bhaskaran M., Sriram S., Kalantar-Zadeh K. (2013). CNT/PDMS composite membranes for H_2_ and CH_4_ gas separation. Int. J. Hydrog. Energy.

[62] Jiang L.Y., Chung T., Kulprathipanja S., Chung N.T.-S. (2006). An investigation to revitalize the separation performance of hollow fibers with a thin mixed matrix composite skin for gas separation. J. Membr. Sci..

[63] Sanip S., Ismail A., Goh P., Soga T., Tanemura M., Yasuhiko H. (2011). Gas separation properties of functionalized carbon nanotubes mixed matrix membranes. Sep. Purif. Technol..

[64] Chung T.-S., Jiang L.Y., Li Y., Kulprathipanja S. (2007). Mixed matrix membranes (MMMs) comprising organic polymers with dispersed inorganic fillers for gas separation. Prog. Polym. Sci..

[65] Bastani D., Esmaeili N., Asadollahi M. (2013). Polymeric mixed matrix membranes containing zeolites as a filler for gas sep-aration applications: A review. J. Ind. Eng. Chem..

[66] Shan M., Seoane B., Andres-Garcia E., Kapteijn F., Gascon J. (2018). Mixed-matrix membranes containing an azine-linked covalent organic framework: Influence of the polymeric matrix on post-combustion CO2-capture. J. Membr. Sci..

[67] Lin R., Hernandez B.V., Ge L., Zhu Z. (2018). Metal organic framework based mixed matrix membranes: An overview on filler/polymer interfaces. J. Mater. Chem. A.

[68] Janakiram S., Ahmadi M., Dai Z., Ansaloni L., Deng L. (2018). Performance of Nanocomposite Membranes Containing 0D to 2D Nanofillers for CO_2_ Separation: A Review. J. Membr. Sci..

[69] Khan M.M., Filiz V., Bengtson G., Shishatskiy S., Rahman M., Lillepaerg J., Abetz V. (2013). Enhanced gas permeability by fabricating mixed matrix membranes of functionalized multiwalled carbon nanotubes and polymers of intrinsic microporosity (PIM). J. Membr. Sci..

[70] Sokhan V.P., Nicholson D., Quirke N. (2004). Transport properties of nitrogen in single walled carbon nanotubes. J. Chem. Phys..

[71] Matranga C., Bockrath B., Chopra N., Hinds B.J., Andrews R. (2006). Raman Spectroscopic Investigation of Gas Interactions with an Aligned Multiwalled Carbon Nanotube Membrane. Langmuir.

[72] Ge L., Zhu Z., Li F., Liu S., Wang L., Tang X., Rudolph V. (2011). Investigation of Gas Permeability in Carbon Nanotube (CNT)−Polymer Matrix Membranes via Modifying CNTs with Functional Groups/Metals and Controlling Modification Location. J. Phys. Chem. C.

[73] Lee R., Jawad Z., Ahmad A., Chua H. (2018). Incorporation of functionalized multi-walled carbon nanotubes (MWCNTs) into cellulose acetate butyrate (CAB) polymeric matrix to improve the CO_2_/N_2_ separation. Process. Saf. Environ. Prot..

[74] Sharma A., Kumar S., Tripathi B., Singh M., Vijay Y. (2009). Aligned CNT/Polymer nanocomposite membranes for hydrogen separation. Int. J. Hydrog. Energy.

[75] Ma P.-C., Siddiqui N.A., Marom G., Kim J.-K. (2010). Dispersion and functionalization of carbon nanotubes for polymer-based nanocomposites: A review. Compos. Part A Appl. Sci. Manuf..

[76] Lin R., Ge L., Diao H., Rudolph V., Zhu Z. (2016). Propylene/propane selective mixed matrix membranes with grape-branched MOF/CNT filler. J. Mater. Chem. A.

[77] Ranjbaran F., Omidkhah M.R., Amooghin A.E. (2015). The novel Elvaloy4170/functionalized multi-walled carbon nanotubes mixed matrix membranes: Fabrication, characterization and gas separation study. J. Taiwan Inst. Chem. Eng..

[78] Swain S.S., Unnikrishnan L., Mohanty S., Nayak S.K. (2017). Carbon nanotubes as potential candidate for separation of H_2_CO_2_ gas pairs. Int. J. Hydrog. Energy.

[79] Mittal G., Dhand V., Rhee K.Y., Park S.-J., Lee W.R. (2015). A review on carbon nanotubes and graphene as fillers in reinforced polymer nanocomposites. J. Ind. Eng. Chem..

[80] Aroon M.A., Ismail A., Montazer-Rahmati M., Matsuura T. (2010). Effect of chitosan as a functionalization agent on the performance and separation properties of polyimide/multi-walled carbon nanotubes mixed matrix flat sheet membranes. J. Membr. Sci..

[81] Eitan A., Jiang K., Dukes D., Andrews A.R., Schadler L.S. (2003). Surface Modification of Multiwalled Carbon Nanotubes: Toward the Tailoring of the Interface in Polymer Composites. Chem. Mater..

[82] Sahoo N.G., Rana S., Cho J.W., Li L., Chan S.H. (2010). Polymer nanocomposites based on functionalized carbon nanotubes. Prog. Polym. Sci..

[83] Raimondo M., Naddeo C., Vertuccio L., Bonnaud L., Dubois P., Binder W.H., Sorrentino A., Guadagno L. (2020). Multifunctionality of structural nanohybrids: The crucial role of carbon nanotube covalent and non-covalent functionalization in enabling high thermal, mechanical and self-healing performance. Nanotechnology.

[84] Merum S., Veluru J.B., Seeram R. (2017). Functionalized carbon nanotubes in bio-world: Applications, limitations and future directions. Mater. Sci. Eng. B.

[85] Bilalis P., Katsigiannopoulos D., Avgeropoulos A., Sakellariou G. (2014). Non-covalent functionalization of carbon nanotubes with polymers. RSC Adv..

[86] Spitalsky Z., Tasis D., Papagelis K., Galiotis C. (2010). Carbon nanotube–polymer composites: Chemistry, processing, mechanical and electrical properties. Prog. Polym. Sci..

[87] Cong H., Zhang J., Radosz M., Shen Y. (2007). Carbon nanotube composite membranes of brominated poly(2,6-diphenyl-1,4-phenylene oxide) for gas separation. J. Membr. Sci..

[88] Sapalidis A.A., Karantzis P.I., Vairis A., Nitodas S.F., Barbe S., Favvas E.P. (2020). A Study of the Reinforcement Effect of MWCNTs onto Polyimide Flat Sheet Membranes. Polymers.

[89] Tseng H.-H., Kumar I.A., Weng T.-H., Lu C.-Y., Wey M.-Y. (2009). Preparation and characterization of carbon molecular sieve membranes for gas separation—the effect of incorporated multi-wall carbon nanotubes. Desalination.

[90] Yu B., Cong H., Li Z., Tang J., Zhao X.S. (2013). Pebax-1657 nanocomposite membranes incorporated with nanoparticles/colloids/carbon nanotubes for CO_2_/N_2_ and CO_2_/H_2_ separation. J. Appl. Polym. Sci..

[91] Moghadassi A.R., Rajabi Z., Hosseini S.M., Mohammadi M. (2013). Preparation and Characterization of Polycarbonate-Blend-Raw/Functionalized Multi-Walled Carbon Nano Tubes Mixed Matrix Membrane for CO_2_ Separation. Sep. Sci. Technol..

[92] Habibiannejad S.A., Aroujalian A., Raisi A. (2016). Pebax-1657 mixed matrix membrane containing surface modified multi-walled carbon nanotubes for gas separation. RSC Adv..

[93] Zhao D., Ren J., Li H., Li X., Deng M. (2014). Gas separation properties of poly(amide-6-b-ethylene oxide)/amino modified multi-walled carbon nanotubes mixed matrix membranes. J. Membr. Sci..

[94] Zhao D., Ren J., Wang Y., Qiu Y., Li H., Hua K., Li X., Ji J., Deng M. (2017). High CO_2_ separation performance of Pebax^®^/CNTs/GTA mixed matrix membranes. J. Membr. Sci..

[95] Sun H., Wang T., Xu Y., Gao W., Li P., Niu Q.J. (2017). Fabrication of polyimide and functionalized multi-walled carbon nanotubes mixed matrix membranes by in-situ polymerization for CO_2_ separation. Sep. Purif. Technol..

[96] Zhang Q., Li S., Wang C., Chang H.-C., Guo R. (2020). Carbon nanotube-based mixed-matrix membranes with supramolecularly engineered interface for enhanced gas separation performance. J. Membr. Sci..

[97] Iranagh F.R., Asghari M., Parnian M.J. (2020). Dispersion engineering of MWCNT to control structural and gas transport properties of PU mixed matrix membranes. J. Environ. Chem. Eng..

[98] Amirkhani F., Mosadegh M., Asghari M., Parnian M.J. (2020). The beneficial impacts of functional groups of CNT on structure and gas separation properties of PEBA mixed matrix membranes. Polym. Test..

[99] Wang D., Zheng Y., Yao D., Yang Z., Xin Y., Wang F., Wang Y., Ning H., Wu H., Wang H. (2019). Liquid-like CNT/SiO_2_ nanoparticle organic hybrid materials as fillers in mixed matrix composite membranes for enhanced CO_2_-selective separation. New J. Chem..

[100] Wong K.C., Goh P.S., Taniguchi T., Ismail A.F., Zahri K. (2019). The role of geometrically different carbon-based fillers on the formation and gas separation performance of nanocomposite membranes. Carbon.

[101] Lia X., Yua S., Lia K., Mab C., Zhanga J., Lia H., Changa X., Zhua L., Xuea Q. (2020). Enhanced gas separation performance of Pebax mixed matrix membranes by incorporating ZIF-8 in situ inserted by multiwalled carbon nanotubes. Sep. Purif. Technol..

[102] Sun H., Gao W., Zhang Y., Cao X., Bao S., Li P., Kang Z., Niu Q.J. (2020). Bis(phenyl)fluorene-based polymer of intrinsic microporosity/functionalized multi-walled carbon nanotubes mixed matrix membranes for enhanced CO_2_ separation performance. React. Funct. Polym..

[103] Li X., Ma L., Zhang H., Wang S., Jiang Z., Guo R., Wu H., Cao X., Yang J., Wang B. (2015). Synergistic effect of combining carbon nanotubes and graphene oxide in mixed matrix membranes for efficient CO_2_ separation. J. Membr. Sci..

[104] Sarfraz M., Ba-Shammakh M. (2018). Harmonious interaction of incorporating CNTs and zeolitic imidazole frameworks into polysulfone to prepare high performance MMMs for CO_2_ separation from humidified post combustion gases. Braz. J. Chem. Eng..

[105] Zhang H., Guo R., Hou J., Wei Z., Li X. (2016). Mixed-Matrix Membranes Containing Carbon Nanotubes Composite with Hydrogel for Efficient CO2 Separation. ACS Appl. Mater. Interfaces.

[106] Comesaña-Gándara B., Chen J., Bezzu C.G., Carta M., Rose I., Ferrari M.C., Sposito E., Fuoco A., Jansen J., McKeown N.B. (2019). Redefining the Robeson upper bounds for CO2/CH4 and CO_2_/N_2_ separations using a series of ul-trapermeable benzotriptycene-based polymers of intrinsic microporosity. Energy Environ. Sci..

